# Cereal based diets modulate some markers of oxidative stress and inflammation in lean and obese Zucker rats

**DOI:** 10.1186/1743-7075-8-27

**Published:** 2011-05-03

**Authors:** Damien P Belobrajdic, Yan Y Lam, Mark Mano, Gary A Wittert, Anthony R Bird

**Affiliations:** 1 Commonwealth Scientific and Industrial Research Organisation (CSIRO) Food Futures Flagship, Adelaide, 5000, Australia; 2 CSIRO Food and Nutritional Sciences, Adelaide, 5000, Australia; 3 Boden Institute of Obesity, Nutrition & Exercise, University of Sydney, Sydney, 2000, Australia; 4 Discipline of Medicine, University of Adelaide, Adelaide, 5000, Australia

## Abstract

**Background:**

The potential of cereals with high antioxidant capacity for reducing oxidative stress and inflammation in obesity is unknown. This study investigated the impact of wheat bran, barley or a control diet (α-cellulose) on the development of oxidative stress and inflammation in lean and obese Zucker rats.

**Methods:**

Seven wk old, lean and obese male Zucker rats (n = 8/group) were fed diets that contained wheat bran, barley or α-cellulose (control). After 3 months on these diets, systolic blood pressure was measured and plasma was analysed for glucose, insulin, lipids, oxygen radical absorbance capacity (ORAC), malondialdehyde, glutathione peroxidase and adipokine concentration (leptin, adiponectin, interleukin (IL)-1β, IL-6, TNFα, plasminogen activator inhibitor (PAI)-1, monocyte chemotactic protein (MCP)-1). Adipokine secretion rates from visceral and subcutaneous adipose tissue explants were also determined.

**Results:**

Obese rats had higher body weight, systolic blood pressure and fasting blood lipids, glucose, insulin, leptin and IL-1β in comparison to lean rats, and these measures were not reduced by consumption of wheat bran or barley based diets. Serum ORAC tended to be higher in obese rats fed wheat bran and barley in comparison to control (p = 0.06). Obese rats had higher plasma malondialdehyde (p < 0.01) and lower plasma glutathione peroxidase concentration (p < 0.01) but these levels were not affected by diet type. PAI-1 was elevated in the plasma of obese rats, and the wheat bran diet in comparison to the control group reduced PAI-1 to levels seen in the lean rats (p < 0.05). These changes in circulating PAI-1 levels could not be explained by PAI-1 secretion rates from visceral or subcutaneous adipose tissue.

**Conclusions:**

A 3-month dietary intervention was sufficient for Zucker obese rats to develop oxidative stress and systemic inflammation. Cereal-based diets with moderate and high antioxidant capacity elicited modest improvements in indices of oxidative stress and inflammation.

## Background

Oxidative stress commonly develops in obesity and has been identified as the unifying mechanism underlying the development of obesity-related co-morbidities. In obesity, a variety of factors contribute to oxidative stress including; hyperglycemia, hyperleptinemia, increased tissue lipid levels, inadequate antioxidant defence, increased rates of free radical formation from enzymatic sources within the endothelium and chronic systemic inflammation [1]. Effective treatments for reducing oxidative stress levels in obese humans include surgical interventions as well as lifestyle modification such as energy restriction and exercise to promote weight loss [1]. In addition, antioxidant rich diets can reduce oxidative stress and inflammatory responses independent of weight loss [2]. Consumption of fruits, vegetables and red wine has been shown to increase serum antioxidant activity *in vivo *in the young and elderly [3-5]. In addition, wholegrains and dietary fibre rich in phenolics and other compounds (β-carotene, iron, manganese, quercetin, tocopherol, zinc and ascorbic acid), also reduce oxidative stress in people with metabolic syndrome [6]. Studies in healthy rodents have shown that phenolic-rich cereals lowered oxidised lipids in blood, liver and brain tissue and increased the activity of antioxidant enzymes in blood including glutathione peroxidase and superoxide dismutase activity [7,8]. However, it is not known whether phenolic-rich cereal based diets reduce oxidative stress in animals with metabolic syndrome.

Diets high in fibre, including cereal fibres are associated with a reduction in inflammatory markers [9,10]. There are a variety of possible mechanisms for this association. As weight loss intervention studies in obese subjects improve the low grade inflammation of subcutaneous and visceral adipose tissue depots [11-13], dietary fibre intervention studies that reduce weight may indirectly reduce inflammation [14]. A psyllium fibre intervention in obese Zucker rats, reduced body weight gain and subsequently reduced circulating tumor necrosis factor (TNF)-α levels [15]. Dietary fibre also reduces markers of inflammation independent of weight loss. During the postprandial response to a meal, cereal fibre elicits a lower rise in plasma interleukin (IL)-6 in comparison to a potato meal [16]. This lower inflammatory response has been attributed to the low glycemic impact of cereal fibre, which if consumed over the long term could contribute to a lower inflammatory status. In addition, cereals high in phenolic compounds may also directly reduce lipid accumulation in adipocytes and pre-adipocytes [17] and have been shown to reduce inflammation in a variety of animal models [18-20].

Zucker obese rats are the most widely used animal model of obesity as they develop dyslipidemia, mild glucose intolerance, hyperinsulinemia, hypertension and low-grade inflammation, manifestations similar to those that define human metabolic syndrome [21,22]. Additionally Zucker obese rats develop oxidative stress. Oxidised lipids are elevated in serum, urine and liver by 14 weeks of age [23-25] and plasma antioxidant defence mechanisms such as glutathione peroxidase are compromised [24,26]. Accordingly the Zucker rat is an appropriate model for studying the effect of dietary components on oxidative stress associated with metabolic disturbances in overweight and obese humans.

This study aimed to compare the effect of diets containing wheat bran (a commonly consumed fibre fraction in Australia) and a wholegrain barley with high *in vitro *antioxidant capacity on markers of oxidative stress and inflammation in lean and obese Zucker rats. It was hypothesised that the cereal diets would increase antioxidant capacity and reduce oxidative stress in obese rats, as well as reduce inflammation in the vascular system and adipose tissue in comparison to a low-antioxidant diet.

## Methods

### Animal and feeding procedures

Male six week old Zucker rats, obese fa/fa (n = 24) and lean Fa/? (n = 24) were obtained from the Flinders University animal house, Bedford Park, South Australia. Rats were housed in wire bottomed cages in a room with controlled heating and lighting (23°C with a 12-h light/dark cycle) and had free access to food and water. After arrival, the rats were adapted to a non-purified commercial diet for one week. They were allocated randomly to three groups (n = 8 per group) and fed one of three diets (Table 1) for twelve weeks.

**Table 1:** Diet composition1

	Control	Wheat bran g/kg	Barley
α-cellulose wheat bran barley	45	164.8	608.1
cornstarch	534.5	447.2	55.3
casein	200	174.1	130.1
sucrose	100	100	100
sunflower seed oil	70	70	70
mineral mix^2^	35	35	35
vitamin mix^3^	10	10	10
cystine	3	3	3
choline	2.5	2.5	2.5

^1 ^All diets contain 4.5% neutral non-starch polysaccharides and the wheat bran and barley were added as a flour of similar particle size (2-3 mm).

^2 ^mineral mix AIN-93G

^3 ^vitamin mix AIN-76, with only 33% of α-tocopherol was provided.

The diets, based on AIN-93G formulation, contained α-cellulose, wheat bran or barley flour which were added to the diet to provide 4.5% neutral non-starch polysaccharide (NNSP) (Table 1). Wheat bran was a commercially available product suitable for human consumption and a conventional barley, cv *Torrens*, was used (supplied by Amanda Box, University of Adelaide). The wheat bran and barley were milled to a particle size of 2-3 mm by the Bread Research Institute, Victoria. Macronutrient composition of the cereals was determined as described previously [27,28] using Official Methods of Analysis of AOAC International [29] (Table 2). For NNSP, a modified version of the gas chromatography method of Theander et al [30] (AOAC method 994.13) was used; the method employed a scaled-down procedure using a 2-h hydrolysis with dilute (1 mol/L) sulphuric acid followed by centrifugation (2000 *g*, 15 min) to obtain the insoluble NNSP, and a further hydrolysis using 2 mol/L trifluoroacetic acid for the soluble NNSP. The vitamin mix was based on AIN-76 [31] with 50 mg α-tocopherol provided per kg of diet which is 33% of the recommended amount. The antioxidant capacity of the diets as determined by the oxygen radical absorbance capacity (ORAC) assay were; Control, 1.1 ± 0.2, wheat bran 7.1 ± 0.1, barley 10.3 ± 0.1 μmol Trolox equivalents/g (Table 2). Food intake was calculated daily (food remaining subtracted from the amount of food provided) for each cage of four animals throughout the twelve weeks feeding study.

**Table 2:** Proximate composition, total phenolics and antioxidant capacity of wheat bran and barley1

	Fat	Protein	Total starch	β-glucan	Total dietary fibre	Neutral non-starch polysaccharides	Phenolics	ORAC^1^
	g/100 g						mg/100 g	μmol Trolox/g
wheat bran	4.1	14.0	15.4	2.0	43.1	24.4	225	83.9
barley	2.5	10.1	64.8	3.0	12.1	6.9	173	33.4

^1^Oxygen radical antioxidant capacity, both lipid and water soluble components were assessed

At the conclusion of the study the rats were fasted overnight (15 h) and then anesthetised with sodium pentobarbital and the femeral artery canulated to enable measurements of blood pressure and heart rate (Biopac data acquisition system, Santa Barbara, CA). Blood was collected from the abdominal aorta, processed to obtain serum and EDTA plasma and stored at -80°C until analysed. The major organs and adipose tissue, including mesenteric, epididymal, retroperitoneal and inguinal fat were removed and weighed. All procedures involving animals were approved by the Commonwealth Scientific and Industrial Research Organisation Food and Nutritional Sciences Animal Ethics Committee.

### Blood analyses

Plasma triglyceride and total cholesterol concentrations were measured in one run on a BM/Hitachi 902 Automatic Analyzer with the use of standard Roche enzymatic kits (Roche Diagnostics Co, Indianapolis, IN). Plasma glucose was measured by YSI 2700 Select Bioanalyser (Yellow Springs, OH), using the 2747 Dual Calibration Standard. The concentrations of insulin, leptin, adiponectin, IL-1β, IL-6, monocyte chemotactic protein (MCP-1), plasminogen activator inhibitor (PAI)-1 and TNF-α in the plasma was determined using a Rat Adipokine multiplex kit (Millipore, St. Charles, MO, USA) according to manufacturer's instructions. The analyses were conducted in a single run and the mean intra-assay coefficient of variation (CVs) ranged from 3-8%. Multianalyte profiling was performed on the LiquiChip 200 Workstation (Qiagen, Melbourne, Australia) and fluorescence data were analysed by using the LiquiChip Analyzer Software (version 1.0.5, Qiagen, Melbourne, Victoria, Australia).

Plasma ORAC was determined according to a modified method of Cao et al. [32], which has been described in detail elsewhere [33]. Briefly, the serum and serum non-protein fractions were extracted by two methods. The ORAC_pca _(perchloric acid) was measured using 1 part serum to 1 part perchloric acid which only extracted water-soluble antioxidants. Following centrifugation, the supernatant was diluted using phosphate buffered saline (1:20). The ORAC_ac _(acetone) was measured using one part serum to nine parts acetone which extracted both lipid and water soluble antioxidants. Following centrifugation, the supernatant was diluted using 7% randomly methylated β-cyclodextrin diluted in one part acetone to one part water (final dilution of serum 1:100). Each serum extract (20 μL), in duplicate, reacted with 25 μL of 3 mM of 2,2'-azobis(2-amidinopropane) 4-hydrochloride at 37°C, and kinetic reads were performed at 60 sec intervals for 50 cycles at an excitation wavelength of 598 nm and an emission wavelength of 615 nm (EnVision, Perkin Elmer, Waltham, Massachusetts). The absorbance readings were plotted against time and the area under the curves was determined. Trolox was used as a reference antioxidant and a standard curve was obtained to calculate the ORAC values for each sample. One ORAC unit was defined as the net protection area provided by 1 μM final concentration of Trolox.

Plasma glutathione peroxidase was analysed using a commercial kit (Sigma, St Louis, Missouri). EDTA plasma was diluted with glutathione peroxidase assay buffer (1:4) and 20 μL was added, in duplicate, to a 96 well plate. Serial dilutions of a glutathione peroxidase standard (Sigma, St Louis, Missouri) were included to obtain a standard curve. Glutathione peroxidase assay buffer (140 μL) was added to each well followed by 10 μL nicotinamide adenine dinucleotide phosphate (NADPH) solution. The plate was transferred to an EnVision plate reader (Perkin Elmer, Waltham, Massachusetts). After 10 min incubation at 25°C, 20 μL t-butyl hydroperoxide was added to each well and kinetic reads were performed at 20 sec intervals for a period of 5 mins at a wavelength of 340 nm. The absorbance readings were plotted against time and the slopes of the linear part of the curves determined. The slope for the blank wells (containing assay buffer, NADPH and t-butyl hydroperoxide) was subtracted from the slope of the lines for standards and plasma samples. The adjusted slopes of the glutathione peroxidase standards were used to generate a standard curve against which the adjusted slopes of the plasma samples were plotted to determine the glutathione peroxidase activity for each sample. The Glutathione peroxidase concentration was reported as U glutathione peroxidase/mL plasma where 1 U of glutathione peroxidase is defined as reducing NADPH at a rate of 1 mmol/mL/min.

**Figure 1:**
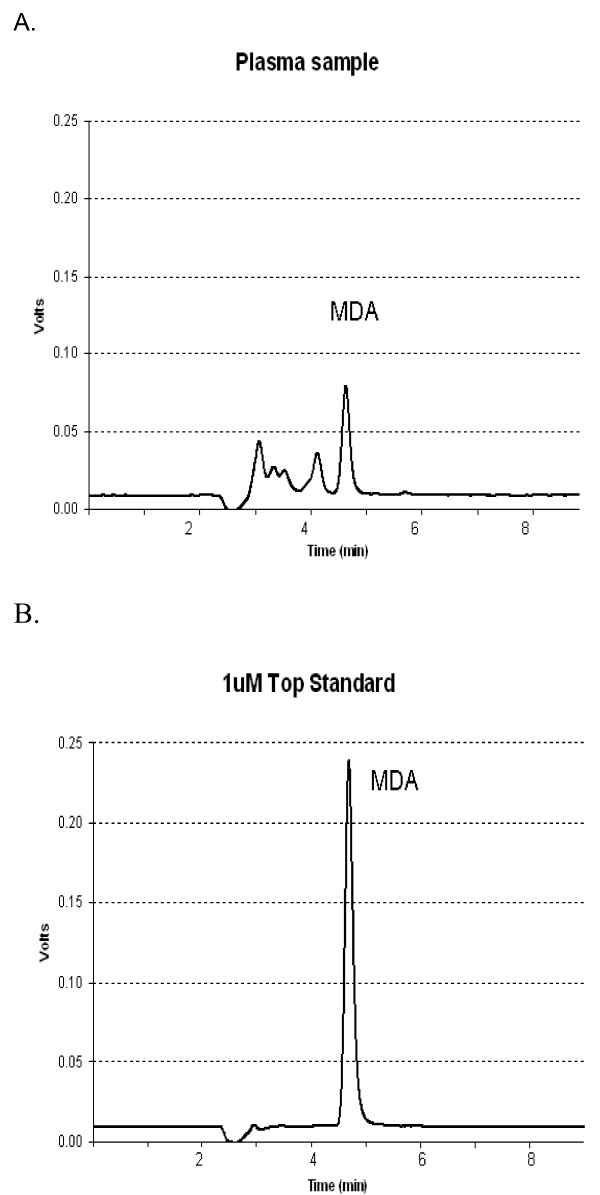
**Typical chromatograms of (A) a plasma sample and (B) 1 μM malondialdehyde (MDA) standard**.

Malondialdehyde was measured in plasma samples by high performance liquid chromatography. A 150 μl aliquot of MDA standard or plasma, in triplicate, was added to polypropylene microfuge tubes kept on ice. The MDA standard was generated from 1,1,3,3-tetraethoxypropane (TEP) by hydrolysis when heated with thiobarbituric acid (TBA) reagent during the assay (1 mole of TEP generates 1 mole of MDA) and a standard curve (0.125 - 1.0 μM) was obtained by diluting the 20 μM TEP standard (stable at 4°C for up to 1 month) in water. To precipitate proteins and release the MDA bound to the amino groups of proteins and other amino compounds [34], 75 μl of cold 1 M HClO_4 _was added to the standard and plasma samples and the tubes were mixed immediately. The tubes were centrifuged at 12,000 g for 3 minutes at 4°C and 150 μL of supernatant transferred into 2 mL glass chromatography vials in a rack on ice. 50 μL of 1 M NaOH was added to all vials and vortex immediately. The fluorescent MDA-TBA derivative was prepared by adding 800 μl TBA reagent (0.25% TBA, 1.25 mM diethylenetriaminepentaacetic acid in 2.5 M acetate buffer pH 3.5) and 10 μL of 5% butylatedhydroxytoluene in ethanol. Samples were mixed, placed in a shaking water bath at 95°C for 60 minutes [35] and immediately cooled in ice water and kept at 7°C prior to analysis by high performance liquid chromatography which consisted of a LC1610 autosampler, LC1150 quarternary pump and WinChrom software (GBC Scientific Equipment Pty Ltd, Braeside, Victoria, Australia). The fluorescence of MDA-TBA was monitored with a Shimadzu RF-10A fluorescence detector (excitation 515 nm and emission: 553 nm). A 40 μL sample was injected onto a Microsorb MV 5 μm C18 reverse phase, 250 × 4.6 mm column (Varian Australia Pty Ltd, Mulgrave, Victoria, Australia) and eluted under isocratic conditions with a mobile phase consisting of 42:58 methanol/50 mM potassium phosphate buffer pH 5.8 at a flow rate of 1.0 mL/minute. A typical chromatogram of a plasma sample and standard are shown in Figure 1.

### Tissue explant experiments

Adipose tissue explants from inguinal (subcutaneous) and peri-renal (visceral) adipose tissue were prepared for tissue culture analysis following the method previously described [36]. The explants were cultured in α-minimal essential medium for 24 h, refreshed, then collected after another 24 h of incubation. The collected media was then filter-sterilised and stored at -80°C until use. The concentrations of adiponectin, IL-1β, IL-6, TNF-α, MCP-1 and PAI-1 (total) in the adipose tissue-conditioned medium were determined using a Rat Adipocyte Linco*plex *Kit (Millipore, St. Charles, MO, USA) according to manufacturer's instructions. Multianalyte profiling was performed on the LiquiChip 200 Workstation (Qiagen, Melbourne, Australia) and fluorescence data were analysed by using the LiquiChip Analyzer Software (version 1.0.5, Qiagen, Melbourne, Victoria, Australia).

### Statistical analyses

Data are presented as the arithmetic mean ± SEM for each treatment group. The effect of diet type and rat phenotype on growth rate was determined by a two-way repeated measures analysis of variance (ANOVA); for all variables two-way ANOVA was performed. Differences between treatments were analysed by a Tukey's post-hoc test. These analyses were performed using SPSS version 17.0 (SPSS Inc., Chicago Il USA). A value of p < 0.05 was taken as the criterion of significance.

## Results

### Food intake and body weight gain

Obese rats consumed 30% more food than lean rats (obese; 33 ± 5, lean; 21 ± 3 g/d, p < 0.001), that contributed to a 43% higher body weight at the end of the study (obese; 609 ± 10, lean; 426 ± 9 g, p < 0.0001). Rats on the wheat bran diet in comparison to those fed the barley diet had a lower weight gain (wheat bran; 25 ± 0.8, barley; 29 ± 0.9 g/wk, p = 0.006) and final body weight (wheat bran; 497 ± 12, barley; 542 ± 12 g, p = 0.034) (Table 3). The Control group did not differ from the wheat bran or barley groups in mean weight gain or final body weight (Table 3).

**Table 3:** Effect of Control, wheat bran and barley based diets on weight gain, organ and tissue weights of lean and obese Zucker rats^1^

	**Lean**			**Obese**						
	**Control**	**Wheat bran**	**Barley**	**Control**	**Wheat bran**	**Barley**	**SEM**	**Phenotype**	**Diet**	P * D
	
Final body weight (g)	420 ^a^	420 ^a^	438 ^a^	612 ^b^	574 ^b^	646 ^b^	16.9	0.0001	0.034	0.244
Weight gain (g/wk)	20.6 ^a^	20.3 ^a^	21.9 ^a^	33.3 ^b^	29.5 ^b^	35.9 ^b^	1.2	0.0001	0.006	0.121
Organ weights (g)										
Liver	9.4 ^a^	10.5 ^a^	10.3 ^a^	21.0 ^c^	18.1 ^bc^	16.7 ^b^	0.77	0.0001	0.109	0.005
Heart	1.11	1.10	1.16	1.17	1.24	1.23	0.034	0.007	0.346	0.554
Kidney	1.1 ^a^	1.1 ^a^	1.2 ^ab^	1.4 ^b^	1.3 ^b^	1.4 ^b^	0.05	0.0001	0.349	0.591
Adipose tissue weights (%BW)^2^	1.7 ^a^	1.6 ^a^	1.7 ^a^	2.6 ^b^	2.8 ^b^	2.4 ^b^	0.2	0.0001	0.757	0.437
Mesenteric	1.9 ^a^	1.9 ^a^	1.9 ^a^	3.0 ^b^	3.4 ^b^	2.9 ^b^	0.2	0.0001	0.398	0.470
Epididymal	2.2 ^a^	2.2 ^a^	2.0 ^a^	5.9 ^b^	5.5 ^b^	5.3 ^b^	0.4	0.0001	0.541	0.852
Retroperitoneal Inguinal	3.2 ^a^	3.3 ^a^	2.9 ^a^	17.6 ^b^	17.9 ^b^	16.4 ^b^	1.5	0.0001	0.805	0.936

^1^A different letter in the same row denotes significant difference, P < 0.05. Data is presented as a mean and pooled SEM, n = 8.

^2 ^%BW, data is expressed as a percentage of rat body weight

### Cardiovascular measures

Obese rats had a slower heart rate (obese; 419 ± 9, lean; 462 ± 8 bpm, p < 0.001) and lower heart weight (obese; 0.20 ± 0.005, lean; 0.26 ± 0.005% of body weight, p < 0.0001) in comparison to lean rats. The obese rats fed with the barley diet had a significantly lower heart rate than the lean rats fed with the Control diet (p < 0.05). Overall, cereal type did not affect heart rate or heart weight. Systolic blood pressure was lower in lean rats than obese rats (lean; 146 ± 5.4, obese; 166 ± 6.0 mmHg, p < 0.05) but was unaffected by cereal type. Diastolic blood pressure was not affected by the obese phenotype or cereal type.

### Adipose tissue and organ weights

The visceral (mesenteric, epididymal and retroperitoneal) and subcutaneous (inguinal) adipose tissue depots were substantially heavier in the obese than lean rats when normalised to body weight (p < 0.0001). Cereal type did not affect adipose tissue weight (Table 3).

Liver weight was lower in lean rats in comparison to obese rats (lean; 10.1 ± 0.4, obese; 18.6 ± 0.5 g, p < 0.0001). In the obese rats, the barley diet fed animals had a lower liver weight than the Control diet fed animals (p < 0.05) (Table 3). There was no effect of diet type on liver weight for lean animals (Table 3). Kidney weights were higher in lean than obese rats (Table 3).

### Serum and plasma metabolites, hormones and adipokines

Lean Zucker rats had lower fasting glucose (lean; 9.7 ± 0.36, obese; 13.1 ± 0.36 mmol/L, p < 0.0001), insulin (lean; 3.9 ± 0.9, obese; 9.7 ± 0.8 ng/mL, p < 0.001), leptin (lean; 9.6 ± 3.6, obese; 45.4 ± 3.3 μg/mL, p < 0.0001) total cholesterol (lean; 2.2 ± 0.2, obese; 5.1 ± 0.2 mmol/L, p < 0.0001), free fatty acids (lean; 0.6 ± 0.3, obese; 2.7 ± 0.3 mmol/L) and triglycerides (lean; 0.16 ± 0.2, obese; 1.5 ± 0.2 mmol/L, p < 0.0001) than obese rats. Cereal type did not affect glucose, insulin, leptin, total cholesterol, free fatty acid or triglyceride concentration.

**Figure 2:**
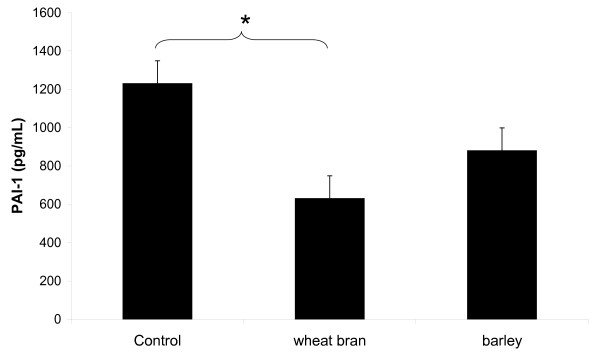
**Effect of control, wheat bran and barley based diets on serum plasminogen activator inhibitor-1 (PAI-1) in obese Zucker rats**. Values are expressed as means ± SEM, n = 8. A significant difference between groups, p < 0.001 is denoted by (*).

Lean rats had lower plasma IL-1β (lean; 112 ± 29, obese; 230 ± 27 pg/mL, p < 0.01) and PAI-1 concentration (lean; 653 ± 88, obese; 913 ± 82 pg/mL, p < 0.05) than obese rats. In the obese rats, the wheat bran diet in comparison to the Control diet, reduced PAI-1 to similar levels as the lean rats (p < 0.05) (Figure 2). IL-6 was unaffected by phenotype or cereal type and TNF-α was not detected in any of the samples (levels were below the detection limit 3.2 pg/mL).

### Antioxidant and oxidised lipid

Obese rats had significantly higher ORAC_ac _than lean rats (obese; 2.2 ± 0.2, lean: 1.0 ± 0.2 μmol Trolox eq/mL, p < 0.0001) (Table 4). In obese rats, wheat bran and barley fed animals tended to have a higher ORAC_ac _than the Control diet fed animals (Table 4, p = 0.064). Phenotype and diet type did not affect ORACp_ca _plasma levels (Table 4).

**Table 4:** Effect of control, wheat bran and barley based diets on serum antioxidant capacity and plasma glutathione peroxidase and malondialdehyde concentration 1

	Lean			Obese						
					
	Control	Wheat bran	Barley	Control	Wheat bran	Barley	SEM	Phenotype	Diet	P * D
ORAC_pca _(μmol Trolox eq/mL)^2^	1.4	1.3	1.3	1.4	1.2	1.1	0.1	0.182	0.204	0.512
ORAC_ac _(μmol Trolox eq/mL)^2^	0.8	1.0	1.1	1.3	2.4	2.8	0.4	0.0001	0.08	0.294
Glutathione peroxidase (μmol/min/ml)	1101	1174	1049	911	980	968	45	0.001	0.274	0.441
Malondialdehyde (nmol/mL)	0.7	0.7	0.7	0.8	1.1	1.0	0.05	0.006	0.472	0.476

^1 ^Data is presented as a mean and pooled SEM, n = 8.

^2 ^Oxygen radical antioxidant capacity assay analysis of serum extracted with perchloric acid (ORAC_pca_) or serum was extracted with acetone (ORAC_ac_).

Obese rats had significantly lower plasma glutathione peroxidase levels in comparison to lean rats (lean; 1108 ± 30, obese; 953 ± 27 μmol/min/mL, p < 0.001). The obese rats fed the wheat bran diet had higher glutathione peroxidase levels than the Control diet-fed obese rats (p < 0.05) (Table 4). There was no difference in plasma glutathione peroxidase between the barley fed animals and the other dietary treatment groups.

Obese rats had significantly higher malondialdehyde levels in comparison to lean (obese; 1.0 ± 0.07, lean; 0.7 ± 0.07 nmol/mL, p < 0.001) (Table 4). Diet type did not affect plasma malondialdehyde levels.

### Adipokine secretory rates from adipose tissue

**Figure 3:**
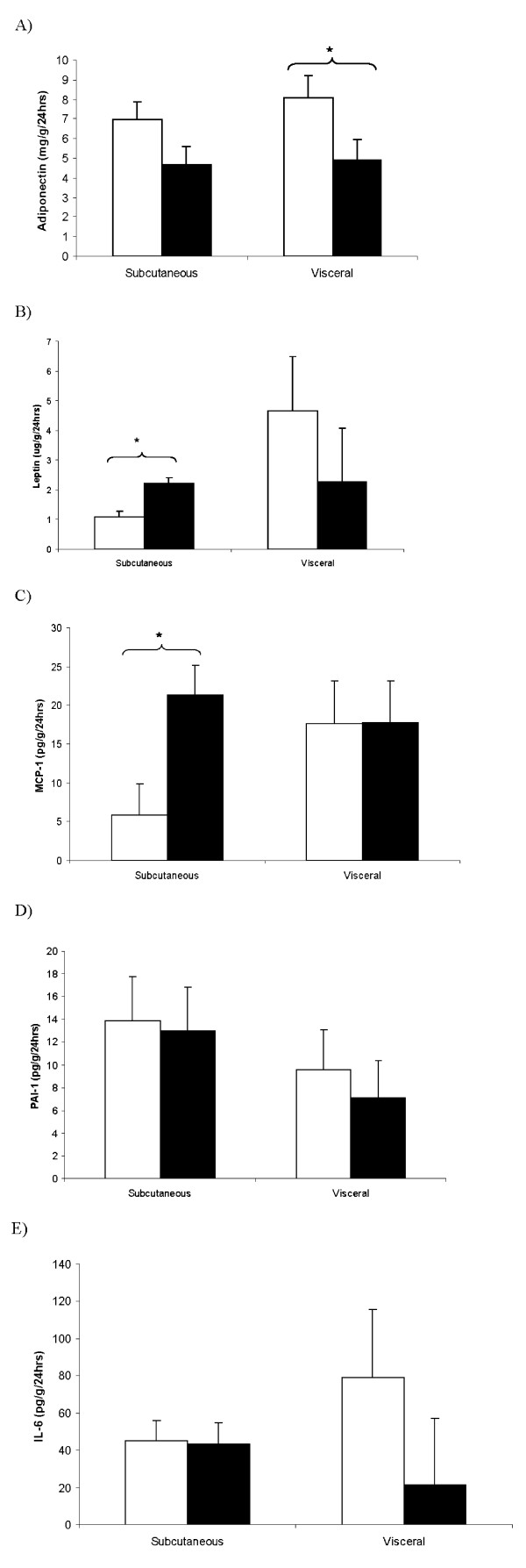
**Adipokine secretion rates from subcutaneous and visceral fat depots in lean (□) and obese (■) Zucker rats**. The concentration of adipokines A) adiponectin, B) leptin, C) MCP-1, D) PAI-1 and E) IL-6 were measured in media that was incubated with adipose tissue for 24 hrs. Values are expressed as means ± SEM, n = 8. A significant difference between groups, p < 0.001 is denoted by (*).

The secretion of adipokines from the subcutaneous fat of obese rats was higher for leptin (p < 0.001) and monocyte chemoatractant protein-1 (MCP-1) (p < 0.01) and a trend to lower adiponectin (p = 0.10) in comparison to lean rats (Figure 3). The secretion of adipokines from the visceral fat of obese rats was lower for adiponectin (p < 0.05), with no change in IL-6, leptin, MCP-1 and PAI-1 (Figure 3). Diet type did not affect adipokine secretion levels from subcutaneous or visceral fat (data not shown).

The secretion of leptin and MCP-1 from subcutaneous (leptin; r = 0.453, p = 0.018, MCP-1; r = 0.447, p = 0.020) but not visceral fat correlated with their circulating levels in plasma. Plasma PAI-1 did not correlate with PAI-1 levels from subcutaneous or visceral fat. Subcutaneous fat secretion of MCP-1 was correlated with plasma levels of insulin (r = 0.456, p = 0.017), leptin (r = 0.524, p = 0.005), and PAI-1 (r = 0.480, p = 0.011).

## Discussion

The purpose of this study was to investigate the impact of wheat bran and barley on markers of oxidative stress and inflammation in lean and obese Zucker rats. In comparison to the Control diet, the wheat bran- and barley-based diets tended to increase the antioxidant capacity of the lipid-soluble antioxidants in the serum. This was evident by the increased antioxidant capacity of serum when extracted by acetone but not perchloric acid. However, the increase in serum antioxidant capacity ORAC_ac _in wheat bran- and barley-fed obese rats did not reduce the amount of oxidised lipids in the plasma, as measured by malondialdehyde, or increase glutathione peroxidase activity. In addition for the lean rats, none of these markers of oxidative stress were affected by the antioxidant capacity of the diet. This is in contrast to a study where rats were fed a barley grain based diet (similar phenolic acid concentration as the current study) which showed that although plasma total antioxidant status remained unchanged, a less specific assay of oxidised lipids in serum, thiobarbituric acid reactive substance, decreased and plasma glutathione peroxidase activity increased [7]. As the analyses were conducted on fasting animals in the current study, the differences between studies could be due in-part to whether or not the animals were fasted. In the fasted state, oxidative stress caused by obesity may override any antioxidant effects that could be achieved by circulating cereal grain components including; phenolics, tocopherols, carotinoids, vitamin C, sterols and phytic acids. To further understand the antioxidant potential of barley *in vivo *it would be important to examine the changes in antioxidant status during the postprandial and post-absorptive stages of digestion in both lean and obese animals. Furthermore, barley cultivars vary in antioxidant capacity. While the barley grain and wheat bran used in the current study had a moderate to high *in vitro *antioxidant capacity, other barley cultivars have been shown to have up to a 2-fold greater antioxidant capacity [37]. Therefore, greater antioxidant effects may be observed if other barley cultivars are used.

In obesity there is an increase in pro-inflammatory cytokine levels in adipose tissue and in the circulation which contributes to oxidative stress. We determined whether cereal-based diets modulate both inflammation and oxidative stress. The wheat bran diet reduced PAI-1 concentration in the plasma but did not affect other adipokine levels; leptin, IL-1β or IL-6. This change in PAI-1 could not be explained by the secretion rates of PAI-1 from subcutaneous or visceral adipose tissue. It is likely that an alternative site of PAI-1 synthesis, the liver, was responsible for regulating circulating PAI-1 levels. Previous studies show that short chain fatty acids produced from the fermentation of dietary fibre in the large intestine inhibit the hepatic synthesis of coagulation factors through inhibition of fatty acid release [38]. In addition, association and cohort studies also show an inverse association between high fibre diets and PAI-1 levels [39-41]. Thus, differences in the fermentation of wheat bran in comparison to barley and α-cellulose may have contributed to the lower PAI-1 levels.

The barley diet had no effect on weight gain or adiposity in lean or obese Zucker rats when compared to the Control diet, even though both diets contained substantial levels of non-starch polysaccharides (4.5%). This is consistent with a study that fed obese Zucker rats an oat bran concentrate-based diet that contained higher levels of β-glucan (10%) [42] than the current study (2%). However, in a different animal model of obesity (diet-induced obese mice), a similar level of dietary β-glucan as provided in the current study (2% β-glucan from barley grain), showed a reduction in weight gain, but not adipose tissue weight [43]. Furthermore, no additional reductions in body weight gain or adiposity were observed with a 4% β-glucan diet. Overall, this suggests that barley and oats (rich in β-glucan) have limited effects on reducing weight gain associated with a genetic model of obesity. However, barley grains may prevent the development of diet-induced obesity by improving insulin sensitivity through altering glucose and lipid metabolism [43]. In addition, other varieties of barley that contain high levels of other types of dietary fibre such as fructan and amylose may provide additional benefits to metabolic health but this deserves further investigation.

In the current study wheat bran did not affect body weight gain or adiposity in lean or obese Zucker rats. Similarly, Neyrinck et al [44] showed that a 10% wheat bran diet did not reduce body weight gain or adiposity compared to a 10% cellulose diet in diet-induced obese mice. The primary non-starch polysaccharide component of wheat bran is arabinoxylan, which is a soluble fibre that is rapidly fermented in the colon. Abarbinoxylan improves lipid and glucose metabolism in humans and rats [45-49], but did not reduce body weight or adiposity when type 2 diabetic patients consumed the fibre fraction for five weeks [50]. Therefore, the potential of wheat bran *per se *in improving metabolic health seems limited.

As the wheat bran and barley grain diets did not reduce adipose tissue weight gain it is likely that the levels of phenolics in these cereals were not sufficient to inhibit lipid accumulation. *In vitro *studies support the direct effect of phenolics on inhibiting lipid accumulation in adipocytes and pre-adipocytes [17]. In particular, the phenolics rutin and o-coumaric acid have been shown have the greatest effect on inhibiting adipogenesis in 3T3-L1 adipocytes [51] and these phenolics when fed to rats, reduced peritoneal and epididymal adipose tissue weights [52]. However, these rats were administered with more than 2.5 fold greater levels of phenolics than provided by the wheat bran or barley diets in the current study.

Even though obese Zucker rats had lower heart rate and weight, and higher systolic and pulse pressure than lean rats, these measures were unaffected by diet type. Son et al [53] reported that rats consuming barley-based high fat diet had larger aortic lumen and thinner wall thickness than rats consuming a rice-based diet, which is suggestive of a reduction in the accumulation of lipid and/or plaque in the cardiovascular system of these animals. They also showed that plasma triglyceride, total and LDL cholesterol was reduced by the barley diet. As the diets contained high levels of fat and cholesterol (20 g/100 g fat provided by soybean oil, beef tallow, lard and corn oil, as well as 1 g/100 g cholesterol) compared to the present study, this may explain why no effect of barley or wheat bran on triglyceride or total cholesterol was seen in the current study.

## Conclusions

Obesity-induced changes in markers related to metabolic syndrome in Zucker rats were similar in animals fed with Control, wheat bran- or barley-based diets. Wheat bran and barley showed modest changes in measures of oxidative stress in obese Zucker rats, but it is not known whether cereal varieties with higher phenolic levels would provide a greater reduction in oxidative stress in these animals. In addition, the wheat bran diet lowered plasma PAI-1 levels in obese rats to levels seen in the lean rats. This reduction in PAI-1 was independent of changes in secretion rates from adipose tissue, thus the effect of a moderate antioxidant diet on the regulation of PAI-1 at other sites such as the liver should be investigated further.

## Abbreviations

ANOVA: Analysis of variance; IL: Interleukin; MDA: Malondialdehyde; MCP-1: monocyte chemotactic protein-1; NNSP: Neutral non-starch polysaccharide; NADPH: Nicotinamide adenine dinucleotide phosphate; ORAC: Oxygen radical absorbance capacity; ORAC_ac_: Oxygen radical absorbance capacity acetone; acid; ORAC_pca_: Oxygen radical absorbance capacity perchloric Plasminogen activator inhibitor-1; PAI-1: 1,1,3,3-tetraethoxypropane; TEP: Thiobarbituric acid; TBA: Tumor necrosis factor; TNFα.

## Competing interests

The authors declare that they have no competing interests.

## Authors' contributions

DPB participated in the design of the study, carried out immunoassays and performed the statistical analyses. ARB participated in the design of the study. YYL participated in the design of the study, carried out the in vitro culture of fat tissue and contributed to data interpretation. MM was responsible for the development of the modified malondialdehyde assay and data interpretation. GAW participated in the design of the study and contributed to data interpretation. All authors contributed to the drafting of the manuscript and agreed on the final version of the manuscript.
